# Correlation between carboxylesterase alleles and insecticide resistance in *Culex pipiens *complex from China

**DOI:** 10.1186/1756-3305-4-236

**Published:** 2011-12-19

**Authors:** Yangyang Liu, Hanying Zhang, Chuanling Qiao, Xiping Lu, Feng Cui

**Affiliations:** 1 Plant Protection College, Shandong Agricultural University, Tai'an, Shandong, 271018 China; 2 State Key Laboratory of Integrated Management of Pest Insects & Rodents, Institute of Zoology, Chinese Academy of Sciences, Beijing, 100101 China

**Keywords:** insecticide resistance, carboxylesterases, mosquito, evolution

## Abstract

**Background:**

In China, large amounts of chemical insecticides are applied in fields or indoors every year, directly or indirectly bringing selection pressure on vector mosquitoes. *Culex pipiens *complex has evolved to be resistant to all types of chemical insecticides, especially organophosphates, through carboxylesterases. Six resistant carboxylesterase alleles (*Ester*) were recorded previously and sometimes co-existed in one field population, representing a complex situation for the evolution of *Ester *genes.

**Results:**

In order to explore the evolutionary scenario, we analyzed the data from an historical record in 2003 and a recent investigation on five *Culex pipiens pallens *populations sampled from north China in 2010. Insecticide bioassays showed that these five populations had high resistance to pyrethroids, medium resistance to organophosphates, and low resistance to carbamates. Six types of *Ester *alleles, *Ester^B1^*, *Ester^2^*, *Ester^8^*, *Ester^9^*, *Ester^B10^*, and *Ester^11 ^*were identified, and the overall pattern of their frequencies in geographic distribution was consistent with the report seven years prior to this study. Statistical correlation analysis indicated that *Ester^8 ^*and *Ester^9 ^*positively correlated with resistance to four insecticides, and *Ester^B10 ^*to one insecticide. The occurrences of these three alleles were positively correlated, while the occurrence of *Ester^B1 ^*was negatively correlated with *Ester^8^*, indicating an allelic competition.

**Conclusion:**

Our analysis suggests that one insecticide can select multiple *Ester *alleles and one *Ester *allele can work on multiple insecticides. The evolutionary scenario of carboxylesterases under insecticide selection is possibly "one to many".

## Background

Mosquitoes, due to their special behavior, physiology and close relationship with humans, act as ideal transmitters of a wide variety of human disease agents, including filariasis, Japanese encephalitis, West Nile virus, dengue and malaria [1]. Chemical insecticides have been extensively used since the 1940s to control mosquito vectors, and four major categories of insecticides have been sequentially applied historically: organochlorines, organophosphates, carbamates and pyrethroids [2-4]. In China, insecticide production is over 3.9 × 10^8 ^kg and application is around 2.5 × 10^8 ^kg annually since 1997; directly or indirectly bringing heavy selection pressure on mosquitoes [5]. As one of seven major species of vector mosquitoes in China, the *Culex pipiens *complex has evolved to be resistant to all types of chemical insecticides, except carbamates, in many regions [5].

Three gene families of detoxification enzymes are involved in metabolic resistance to chemical insecticides: carboxylesterases, P450 monooxygenases, and glutathione S-transferases [6]. Carboxylesterases often mediate resistance to organophosphates, carbamates, and to a lesser extent, pyrethroids [1]. In mosquitoes, non-specific carboxylesterases sequester, rather than rapidly metabolize, the pesticide molecules [7]. Two gene loci, *Est-2 *and *Est-3*, encode non-specific esterases through gene amplification or up-regulation in the *C. pipiens *complex [8,9]. These loci are always in complete linkage disequilibrium and referred to as the *Ester *superlocus. To date, twelve *Ester *alleles conferring OP resistance have been identified at the *Ester *locus (the corresponding overproduced esterases are named in parentheses): *Ester^1 ^*(A1), *Ester^2 ^*(A2-B2), *Ester^4 ^*(A4-B4), *Ester^5 ^*(A5-B5), *Ester^8 ^*(A8-B8), *Ester^9 ^*(A9-B9), *Ester^11 ^*(A11-B11), *Ester^B1 ^*(B1), *Ester^B6 ^*(B6), *Ester^B7 ^*(B7), *Ester^B10 ^*(B10) and *Ester^B12 ^*(B12) [5,9-11].

An unusual diversity of *Ester *alleles was observed in field populations from China, where *Ester^B1^, Ester^2^, Ester^B6^, Ester^B7^, Ester^8^, Ester^9^, Ester^B10 ^*and *Ester^11 ^*were reported, and as many as six *Ester *alleles co-existed in one population [12]. This situation is atypical, and may represent a complex situation for the evolution of insecticide resistance genes in China. Accumulation and analysis of monitoring data will be helpful to understand the evolution of insecticide resistance genes in field mosquito populations.

In recent years, we monitored the resistance levels and *Ester *phenotype frequencies of *C. pipiens *complex field populations to organophosphates, carbamates, and pyrethroids at either the large scale or locally in China [13]. In order to explore the evolutionary scenario of *Ester *resistance alleles, we analyzed the data from five *Culex pipiens pallens *populations sampled in north China, and from an historical record from 2003 [12]. We investigated the resistance levels of these five populations towards three organophosphate, two pyrethroid, and two carbamate insecticides. Esterase phenotype frequencies of *Ester *locus were also quantified. Lastly, we combined our data with that of a large scale investigation from 2003 [12], and statistically analyzed the relationship between resistance level and *Ester *phenotype frequency for each insecticide, and among phenotype frequencies of various *Ester *alleles.

## Methods

### Mosquito samples and strains

Five field populations of *C. p. pallens *were collected as egg-rafts, larvae, or pupae in north China from July to August of 2010 (Table 1). They were raised for several generations in the laboratory at 25 ± 1° and a photoperiod of 14L : 10D. Several standard strains were used as references in bioassays or starch gel electrophoresis: S-LAB, an insecticide-susceptible strain without any known resistance genes [14]; SB1 strain, homozygous for *Ester^B1^*, displaying overproduced esterase B1; SA2 strain, homozygous for *Ester^2^*, displaying overproduced esterases A2-B2 [15]; MAO2 strain, homozygous for *Ester^8^*, displaying overproduced esterases A8-B8 [16]; LING strain, homozygous for *Ester^9^*, displaying overproduced esterases A9-B9 [16]; KARA2 strain, homozygous for *Ester^B10^*, displaying overproduced esterase B10 [5]; and WU strain, homozygous for *Ester^11^*, displaying overproduced esterases A11-B11 [5].

**Table 1:** Collection information of *Culex pipiens pallens *sampled in China.

Province	Locality (latitude, longitude)	Code	Date	Type of sites
Henan	Weihe (35°02' N, 113°8' E)	WHE	15/07/2010	sewage tank
	Yuanyang (35°02' N, 113°58' E)	YUY	14/07/2010	cesspool
Shandong	Taian (35°38' N, 116°2' E)	TAA	24/07/2010	puddle
Beijing	Beijing (39°54' N, 116°28' E)	BJI	29/07/2010	sewage
Liaoning	Pulandian (39°23' N, 121°58' E)	LPU	20/07/2010	puddle

### Insecticide bioassays

Resistance characteristics of larvae were determined by bioassays on fourth-instar larvae, following the methods described in Raymond and Marquine (1994) [17]. The test insecticides were three organophosphates, dichlorvos, fenitrothion, malathion, two pyrethroids, deltamethrin, permethrin, and two carbamates, propoxur, BPMC (2-sec-Butylphenyl methyl carbamate). Four or five doses and four replicates (20 larvae per replicate) per dose were performed with each insecticide. The mortalities of larvae were recorded after 24 hours of treatment. Larvae from S-LAB strain were tested at the same time as susceptible controls. Based on Finney (1971) [18] data were analyzed by the log-probit program of Raymond (1993) [19], which provides LCs, slope for each mortality line, parallelism between different mortality lines and resistance ratios with 95% confidence intervals.

### Starch gel electrophoresis

*Ester *allele phenotypes of individual adult mosquitoes were revealed using starch gel electrophoresis (TME 7.4 buffer system) as described by Pasteur et al. (1981; 1988) [20,21]. Mosquitoes from six standard strains (SB1, SA2, MAO2, LING, KARA2 and WU) were used as markers in electrophoresis to indicate the esterase phenotypes of field collected mosquitoes.

### Statistical analysis of correlation

The data of 5 populations from this study and 20 populations from the investigation in 2003 [12] were combined for correlation analysis between resistance levels of insecticides (dichlorvos, parathion, chlorpyrifos, propoxur and BPMC) and *Ester *phenotype frequencies and among phenotype frequencies of various *Ester *alleles using SPSS 13.0.

## Results

### Insecticide resistance status of field populations

Five field populations of *C. p. pallens*, collected in north China (Table 1), were tested for resistance levels towards frequently applied organophosphate, pyrethroid, and carbamate insecticides with larvae bioassays. The tests were finished within three generations (F1-F3) after the mosquitoes were brought back and raised in the lab. Bioassay results indicate that the five populations showed highest resistance levels towards pyrethroids, followed by organophosphates, and last to carbamates (Table 2). For the two pyrethroid insecticides, resistance to permethrin was higher than to deltamethrin. Most of the populations had a resistance ratio (RR) higher than 10 to permethrin, with the highest reaching 68 folds in the population TAA, while lower than 10 to deltamethrin. For the three organophosphate insecticides, several populations showed medium resistance (RR between 10 and 20) to dichlorvos, while for fenitrothion and malathion only lower than 4-fold resistance was observed. All the populations had low resistance (RR lower than 10) to the two carbamate insecticides, propoxur and BPMC.

**Table 2:** Resistance observed in bioassays to seven insecticides in five populations of *Culex pipiens pallens *from China.

Insecticides	Populations	N	LC50 (95% CI) (mg/L)	Slope (SE)	χ^2^	RR (95% CI)	G
Dichlorvos	S-LAB	300	0.19 (0.18-0.19)	13.8 (1.6)	1.7	1	F2
	WHE	240	3.04 (2.86-3.17)	8.0 (1.3)	4.8	16.4 (12.3-21.7)	F1
	YUY	240	3.86 (3.22-4.07)	11.1 (3.4)	0.5	20.8 (13.4-32.3)	F1
	TAA	300	2.91 (2.11-3.27)	5.2 (1.4)	0.4	15.7 (10.2-24.3)	F2
	BJI	300	1.26 (1.19-1.33)	9.7 (1.3)	1.6	6.8 (4.9-9.4)	F2
	LPU	300	1.06 (0.88-1.14)	7.4 (2.3)	1.6	5.7 (3.9-8.3)	F2
Fenitrothion	S-LAB	300	0.0078 (0.0076-0.0081)	15.8 (1.8)	5.2	1	F2
	WHE	300	0.015 (0.014-0.016)	6.8 (1.6)	2.3	1.9 (1.4-2.6)	F1
	YUY	360	0.0099 (0.0084-0.011)	4.0 (0.9)	0.4	1.3 (0.9-1.7)	F1
	TAA	240	0.020 (0.018-0.022)	6.6 (1.7)	1.5	2.5 (1.8-3.6)	F1
	BJI	300	0.0096 (0.0088-0.010)	5.6 (0.7)	0.8	1.2 (0.9-1.6)	F2
	LPU	300	0.015 (0.014-0.016)	6.9 (1.0)	0.6	1.9 (1.4-2.6)	F2
Malathion	S-LAB	240	0.025 (0.020-0.030)	12.0 (4.1)	7.3	1	F2
	WHE	360	0.094 (0.089-0.099)	7.4 (0. 8)	2.2	3.8 (1.8-8.0)	F2
	YUY	360	0.073 (0.069-0.076)	6.8 (1.6)	2.3	3.0 (1.4-6.2)	F1
	TAA	300	0.080 (0.073-0.086)	6.9 (1.5)	3.0	3.3 (1.4-7.8)	F1
	BJI	300	0.073 (0.066-0.081)	4.1 (0.6)	3.9	3.0 (1.4-6.3)	F2
	LPU	240	0.052 (0.044-0.062)	3.7 (0.6)	1.4	2.1 (0.9-5.2)	F2
Deltamethrin	S-LAB	300	0.00039 (0.00036-0.00043)	5.3 (1.1)	0.3	1	F2
	WHE	360	0.0026 (0.0021-0.0029)	4.1 ( 0.9 )	6.0	6.6 (4.9-8.7)	F2
	YUY	360	0.0046 (0.0022-0.0054)	3.8 ( 1.3 )	0.05	11.7 (7.4-18.1)	F1
	TAA	240	0.0039 (0.0029-0.0046)	2.3 (0.5)	0.4	9.9 (7.4-12.9)	F2
	BJI	300	0.0023 (0.0020-0.0024)	5.8 (1.4)	0.004	5.8 (4.3-7.6)	F2
	LPU	240	0.0014 (0.0007-0.0017)	3.8 (1.1)	0.01	3.5 (2.1-5.9)	F2
Permethrin	S-LAB	300	0.0017 (0.0016-0.0019)	5.6 (0.6)	4.8	1	F2
	WHE	300	0.038 (0.035-0.041)	9.4 (1.8)	0.4	21.9 (15.6-30.5)	F2
	YUY	300	0.055 (0.043-0.062)	3.8 (0.9)	2.1	31.9 (22.9-43.9)	F1
	TAA	300	0.12 ( 0.04-0.17 )	1.4 (0.4)	0.2	68.2 (46.9-99.0)	F2
	BJI	360	0.024 (0.023-0.025)	11.0 (1.8)	2.0	13.8 (10.3-18.5)	F2
	LPU	240	0.016 (0.011-0.021)	2.0 (0.6)	0.4	9.5 (6.9-12.9)	F3
Propoxur	S-LAB	300	0.13 (0.13-0.14)	9.7 (1.4)	0.6	1	F2
	WHE	300	0.28 (0.26-0.31)	7.1 (1.6)	1.2	2.1 (1.5-2.9)	F2
	YUY	300	0.18 (0.09-0.21)	4.9 (1.6)	1.6	1.3 (0.8-2.3)	F1
	TAA	300	0.27 (0.25-0.28)	9.7 (1.4)	0.2	2.0 (1.5-2.7)	F2
	BJI	300	0.23 (0.21-0.25)	8.5 (2.1)	0.1	1.8 (1.3-2.5)	F2
	LPU	300	0.21 (0.19-0.23)	6.5 (2.0)	0.3	1.6 (1.2-2.1)	F2
BPMC	S-LAB	300	0.097 ( 0.025-0.35 )	10.8 (3.8)	6.2	1	F2
	WHE	360	0.39 (0.36-0.42)	7.7 (2.1)	3.5	4.0 (0.9-17.7)	F2
	YUY	240	0.23 (0.17-0.26)	3.5 (0.7)	4.5	2.3 (0.8-7.2)	F1
	TAA	240	0.42 (0.39-0.44)	12.7 (2.6)	0.2	4.3 (1.0-19.2)	F2
	BJI	300	0.34 (0.31-0.35)	8.9 (1.4)	0.4	3.4 (1.2-10.2)	F2
	LPU	300	0.21 (0.16-0.24)	3.9 (1.0)	0.2	2.1 (0.5-9.6)	F3

S-LAB, the susceptible reference strain. N, number of larvae tested. CI, confidence interval. RR, resistance ratio (LC_50 _of the population/LC_50 _of S-LAB). G, the generation considered in bioassays.

Populations from Henan (WHE and YUY) and Shandong (TAA) provinces were more resistant to insecticides than those from Beijing (BJI) and Liaoning (LPU) province. BJI and LPU showed low levels of resistance to all tested insecticides. Populations WHE, YUY, and TAA had medium resistance to dichlorvos, and high resistance (RR higher than 20) to permethrin.

### Identification of esterase phenotypes

**Figure 1:**
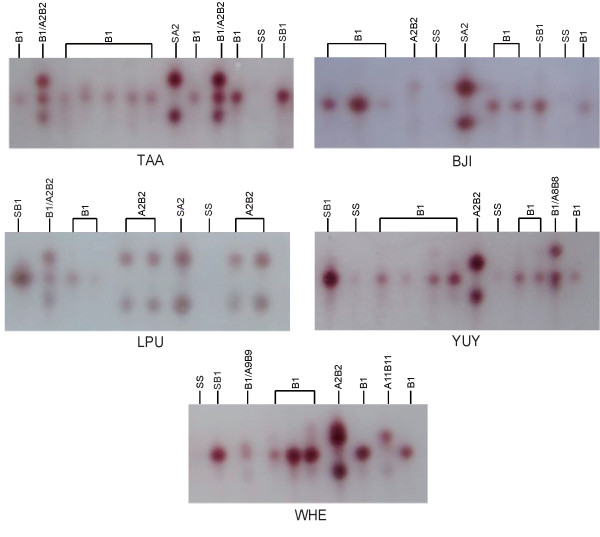
**High-activity esterases in single adult mosquitoes from the five populations analyzed in starch electrophoresis**. High-activity esterases in single adult mosquitoes from the five populations analyzed in starch electrophoresis. Only part of the gel for each population was shown. SB1 and SA2 were the standard strains displaying overproduced esterase B1 and A2-B2, respectively. SS means susceptible individuals.

In order to detect the types of high active carboxylesterases, at least thirty individual adult mosquitoes of each population were applied to starch gel electrophoresis. It has been reported that these high active carboxylesterases are expressed in both adults and larvae [22,23]. A total of 200 adult mosquitoes were checked for the five populations (Table 3). Six types of overproduced esterases were identified in this investigation, i.e. B1, A2-B2, A8-B8, A9-B9, B10, and A11-B11. In the populations from Henan province (WHE and YUY), all six types of esterases were detected, and four esterases co-existed in one population, WHE. In the other three populations, only B1 and A2-B2 were detected (Figure 1). B1 was the predominant esterase, with frequencies over 70 % in most populations. Although the frequencies of A2-B2 were not as high as B1, it still had a distribution as broad as B1. Esterases A8-B8, A9-B9, B10 and A11-B11 were minor, with limited distribution and low frequencies.

**Table 3:** Frequency* of mosquitoes displaying a given overproduced esterase in field populations of *Culex pipiens pallens *in China

Population code	N	B1	A2-B2	A8-B8	A9-B9	B10	A11-B11	SS
WHE	35	0.83	0	0	0.09	0.06	0.03	0.06
YUY	41	0.88	0.2	0.05	0	0	0	0.17
TAA	45	0.98	0.04	0	0	0	0	0.02
BJI	36	0.72	0.03	0	0	0	0	0.25
LPU	43	0.56	0.14	0	0	0	0	0.33

* The sum of phenotypic frequency in each population is not necessarily equal to 1, considering some individuals are heterozygous with two overproduced esterases.

N, the sample size analyzed. SS, the non-overproduced esterase phenotype.

### Correlation analysis

**Figure 2:**
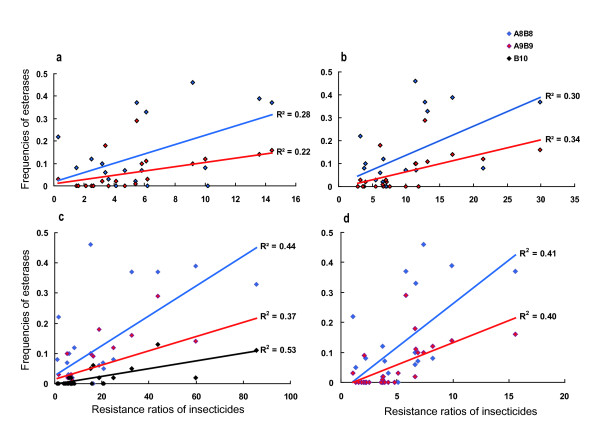
**Correlations between esterase frequencies and resistance ratios to chlorpyrifos**. Correlations between esterase frequencies and resistance ratios to chlorpyrifos (a), parathion (b), dichlorvos (c) and propoxur (d).

Correlation analyses between insecticide resistance levels (dichlorvos, parathion, chlorpyrifos, propoxur and BPMC) and *Ester *phenotype frequencies showed that the frequencies of esterases A8-B8 and A9-B9 positively correlated to the resistance levels of dichlorvos, parathion, chlorpyrifos and propoxur. Esterase B10 only positively correlated to dichlorvos resistance, even though the correlation coefficients (R^2^) were not high (Figure 2). None of the six types of esterases displayed a linear correlation to the BPMC resistance in mosquitoes. Frequencies of esterases B1, A2-B2, and A11-B11 did not linearly correlate to any resistance of the five insecticides. For each insecticide, except for BPMC, multiple types of esterases had a positive relationship with its resistance in mosquitoes.

**Figure 3:**
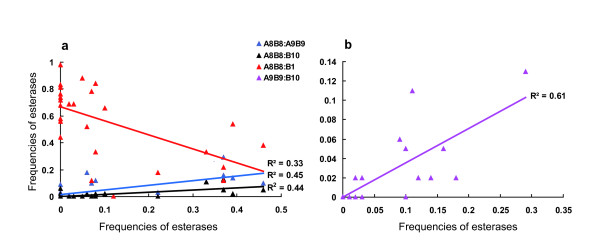
**Correlations among frequencies of various esterases**. Correlations among frequencies of various esterases. (a) between A8-B8 and A9-B9, A8-B8 and B10, and A8-B8 and B1; (b) between A9-B9 and B10.

The correlation among phenotype frequencies of various *Ester *alleles was also checked. The results demonstrate that there were positive correlations between phenotype frequencies of A8-B8 and A9-B9, A8-B8 and B10, A9-B9 and B10, and a negative correlation between A8-B8 and B1 (Figure 3). The existence of A2-B2 or A11-B11 had no linear relationship with other esterases.

## Discussion

As a periodic investigation, we surveyed the resistance levels of five field populations of *C. p. pallens *towards seven insecticides commonly used in mosquito control in China. High and medium resistance appeared to pyrethroids and organophosphates, in some populations, respectively, and only low resistance was observed for carbamates in the five populations. Compared with previous investigations in 2003 [12] and 2006 [13], there is no remarkable change in the organophosphate and carbamate resistance status in the 2010 survey. The resistance to dichlorvos is still leading in organophosphate resistance. Low resistance to the two carbamate insecticides implies that there is still no target resistance mediated by G119S mutation in the acetylcholinesterase 1. On the other hand, the pyrethroid resistance seems elevated, especially the resistance to permethrin. Most of the populations in this survey had the permethrin resistance ratio higher than 10, including the Beijing population BJI, while the other four Beijing populations surveyed in 2006 were susceptible or only showed resistance below 2 fold towards permethrin and tetramethrin [13]. This is the aftermath of an increase of pyrethroid insecticides applied in recent years in China.

In this study we observed six highly active carboxylesterases in these northern populations. The overall pattern of their frequencies in geographic distribution is consistent with the reports seven years prior to this study [5,12]. Henan province still has impressive diversity of *Ester *alleles. Shandong and Liaoning provinces also only have *Ester^B1 ^*and *Ester^2^*. This means the *Ester *alleles did not extend their geographic range during the seven years in these areas. In the Beijing population, where four *Ester *alleles were recorded previously, only *Ester^B1 ^*and *Ester^2 ^*were observed. One possible reason for this difference could be that our sample size in the Beijing region was not large enough. More attention should be given to this region in future.

Among the six types of *Ester *alleles, *Ester^8 ^*and *Ester^9 ^*have linear correlation with resistance to four organophosphate or carbamate insecticides and *Ester^B10 ^*to one organophosphate insecticide in field populations. These three alleles are adaptive to the selection pressure endemic to China, and the occurrences of these alleles are positively correlated at a certain coefficient. In contrast, two ubiquitously distributed alleles around the world, *Ester^B1 ^*and *Ester^2^*, do not show linear correlation with resistance to any one of the five insecticides, implying that they play a general role in organophosphate and carbamate resistance. Although the endemic allele *Ester^11 ^*did not linearly correlate with resistance to the five insecticides, this can not rule out the possible correlation of *Ester^11 ^*with resistance to malathion, fenitrothion, or other insecticides used in China.

Our results suggest that one insecticide can select multiple *Ester *alleles and one *Ester *allele can work on multiple insecticides. So the evolutionary scenario of carboxylesterases under insecticide selection in the field is most likely "one to many", not "one to one". The relationship of *Ester^B1 ^*and *Ester^8 ^*is very interesting. Their occurrence seems to contradict each other. This allelic competition was illustrated by the situation in southern France, where *Ester^1 ^*had been replaced by *Ester^4 ^*over a 10 year period [24], and then followed by the local occurrence of *Ester^2 ^*[25]. It is likely that, in the future, as the result of the allelic competition, *Ester^B1 ^*will be eliminated from regions predominated by *Ester^8^*, such as the Guangdong province, and *Ester^8 ^*will be prevented from invading, or eliminated from, regions predominated by *Ester^B1 ^*such as Beijing, and the Shandong province. Future work is needed to identify the parameters driving the competition between these two alleles.

## Conclusion

Our analysis suggests that one insecticide can select multiple *Ester *alleles and one *Ester *allele can work on multiple insecticides. The evolutionary scenario of carboxylesterases under insecticide selection is possibly "one to many". This study will shed light on the understanding of the evolution of insecticide resistance genes in field populations and guide the management of insecticide resistance.

## Competing interests

The authors declare that they have no competing interests.

## Authors' contributions

YL and HZ performed the study, analyzed the data and drafted the manuscript. CQ supervised the study and helped draft the manuscript. XL and FC conceived and designed the study, and helped draft the manuscript. All authors approved the final version of the manuscript.
